# Acquisition of fluoroquinolone resistance leads to increased biofilm formation and pathogenicity in *Campylobacter jejuni*

**DOI:** 10.1038/s41598-019-54620-1

**Published:** 2019-12-03

**Authors:** Matthew V. X. Whelan, Laura Ardill, Kentaro Koide, Chie Nakajima, Yasuhiko Suzuki, Jeremy C. Simpson, Tadhg Ó Cróinín

**Affiliations:** 1 School of Biomolecular and Biomedical Science, University College Dublin, Belfield, Dublin 4 Ireland; 2 Division of Bioresources, Hokkaido University Research Center for Zoonosis Control, Sapporo, Japan; 3 The Global Station for Zoonosis Control, Hokkaido University Global Institution for Collaborative Research and Education, Kita 20 Nishi 10, Kita-ku, Sapporo, Japan; 4 School of Biology and Environmental Science, University College Dublin, Belfield, Dublin 4 Ireland

**Keywords:** Antimicrobial resistance, Biofilms

## Abstract

The World Health Organization has listed *C*. *jejuni* as one of 12 microorganisms on a global priority list for antibiotic resistance due to a rapid increase in strains resistant to fluoroquinolone antibiotics. This fluoroquinolone resistance is conferred through a single point mutation in the QRDR region within the *gyrA* gene known to be involved in DNA supercoiling. We have previously revealed that changes in DNA supercoilikng play a major role in the regulation of virulence in *C*. *jejuni* with relaxation of DNA supercoiling associated with increased attachment to and invasion of human epithelial cells. The aim of this study was to investigate whether fluoroquinolone resistant strains of *C*. *jejuni* displayed altered supercoiling associated phenotypes. A panel of fluoroquinolone resistant mutants were derived and shown to have a greater ability to form viable biofilms under aerobic conditions, invade epithelial cells and promote virulence in the *Galleria mellonella* model of infection. We thus report for the first time that fluoroquinolone resistance in *C*. *jejuni* is associated with an increase in virulence and the ability to form viable biofilms in oxygen rich environments. These altered phenotypes likely play a critical role in the continued increase in fluoroquinolone resistance observed for this important pathogen.

## Introduction

*Campylobacter jejuni* is the leading cause of bacterial gastroenteritis and a significant health burden across the world^1^. Although the organism is thought to exist as a commensal in the intestinal tract of chickens it becomes highly invasive upon colonization of the human intestinal tract causing severe but usually self-limiting gastroenteritis^2^. The organism is a microaerophilic bacteria which requires a reduced oxygen environment to grow. However the organism appears to have an ability to survive for long periods of time in the presence of oxygen such as on the carcass of a chicken in the supermarket. This ability for the bacteria to survive in the presence of atmospheric levels of oxygen may be a critical factor which enables contaminated poultry meat to function as an important reservoir of infection for this pathogen^3–5^.

Fluoroquinolone antibiotics are broad spectrum antibiotics which are routinely used to treat undiagnosed diarrhoeal infections as well as being used in some countries to treat animals during intensive production^6,7^. Recent studies have revealed a dramatic increase in the number of fluoroquinolone resistant (FQ^R^) strains of *C*. *jejuni* with the Centres for Disease Control and Prevention (CDC) revealing that between 1997 and 2015 an 8.55% increase in the number of ciprofloxacin resistant strains was observed^8,9^. In addition, the World Health Organization recently listed *C*. *jejuni* as one of 12 priority pathogens due in part to this increase in the prevalence of fluoroquinolone resistance^10^. Fluoroquinolones work by inhibiting the function of the DNA Gyrase heterodimer GyrAB and high level fluoroquinolone resistance can be obtained by acquisition of a single point mutation in the QRDR region within the *gyrA* gene of *C*. *jejuni*^11–13^. The predominant mutation associated with resistance is a C257T mutation resulting in a threonine to isoleucine amino acid substitution (Thr86Ile) although other less common mutations have been observed such as Thr86Ala, Thr86Lys, Asp90Asn and Asp90Tyr. To date no mutations in the *gyrB* gene have been associated with fluoroquinolone resistance in *C*. *jejuni* and although the CmeABC multidrug efflux system has also been implicated in intrinsic resistance to fluoroquinolones, mutations in this system have been reported to lead to an increase in fluoroquinolone susceptibility^14–16^. Previous studies of fluoroquinolone resistant mutants in *C*. *jejuni* have suggested that in addition to providing a defence mechanism against the antibiotic these mutations may deliver fitness benefits during the commensal colonization of chickens^17^. Although both fluoroquinolone sensitive and resistant strains colonised chickens efficiently, when co-infection studies were carried out the fluoroquinolone resistant population out competed the sensitive population within three days^17^. The study also revealed that some fluoroquinolone mutations in *gyrA* could result in changes in resting DNA supercoiling levels and this was confirmed in a later study by Han *et al*.^18^.

DNA supercoiling has long been known to play a key role in the regulation of virulence for a variety of pathogens^19–22^. In *C*. *jejuni* DNA supercoiling is predominantly controlled through the activity of the DNA Gyrase heterodimer GyrAB and DNA Topoisomerase 1 (TopA). Recent studies from our group have revealed a key role played by DNA supercoiling in the regulation of virulence factors by *C*. *jejuni* and in particular in the transition from a more commensal to a more invasive phenotype^23,24^. Strains with greater supercoiling activity have been shown to be more motile and this increase in motility was revealed to be induced by the presence of chicken gastrointesintal mucus and was dependent on the source of mucus from within the gastrointestinal tract^24^. Conversely, strains with reduced DNA supercoiling and thus more relaxed DNA were found to be less motile. Furthermore, by using subinhibitory concentrations of novobiocin to artificially relax DNA supercoiling, highly motile strains could be made less motile^24^. Relaxation of DNA supercoiling also made the strains more invasive showing that DNA supercoiling plays a critical role in the global regulation of this transition from a non-invasive to invasive phenotype^23^. Thus although motilty has been shown to play a role in invasion in several studies^25,26^ it appears that relaxation of DNA supercoiling leads to a decrease in motility but an increase in invasion. Interestingly, relaxation of DNA also induced protein secretion that appeared to be reliant on an intact flagella supporting previous reports that the flagella plays a key role in secretion of virulence factors by *C*. *jejuni*^27,28^. Our hypothesis to date is thus that relaxation of DNA supercoiling changes the behaviour of the bacteria from a more motile to a more invasive phenotype and that the flagella is critical for both.

As previously stated, one of the key environments for *C*. *jejuni* to contend with is that of atmospheric oxygen to allow transmission to new hosts, and particularly in foodborne transmission from poultry meat to humans. The mechanism by which *C*. *jejuni* survives in this external environment is poorly understood as the organism lacks many of the stress response genes found in other bacteria but it is known that the secretion of a variety of oxidative stress response proteins have been demonstrated to be critical for biofilm formation in *C*. *jejuni*^29–31^. The previously reported observation that relaxation of DNA supercoiling leads to the production of a specific secretome which aids in invasion^23^ raises the question as to whether this specific secretome could also play a role in the production of the extracellular matrix of the biofilm. Furthermore, the effect of DNA supercoiling on the expression of flagellar genes as well as the putative role of the flagella in secretion could also signal a possible role in biofilm formation given that studies have highlighted a role for flagella in biofilm production^30,32^. The increased incedence of fluoroquinolone resistant strains of *C*. *jejuni* isolated from chicken meat even in countries which do not allow for large scale use of these antibiotics in livestock suggests that resistance to fluoroquinolones may confer a transmission advantage to *C*. *jejuni* beyond protection from antibiotics. Together, these observations raise the prospect that resistance to fluoroquinolone antibiotics affects DNA supercoiling in a way that could lead to changes in virulent phenotypes and could possibly affect the ability of the bacteria to survive in the external environment.

The aim of this study was thus to investigate whether clones selected for fluoroquinolone resistance would have an increased ability to form biofilm in the presence of oxygen as well as testing whether these clones had altered DNA supercoiling levels. Furthermore the effect of fluoroquinolone resistance on the virulence of *C*. *jejuni* would be tested using a variety of *in vitro* and *in vivo* assays.

## Materials and Methods

### Bacterial strains and growth conditions

*C*. *jejuni* NCTC11168 is the type strain of *C*. *jejuni* and *C*. *jejuni* 81-176 is a commonly used laboratory strain which has been previously shown to have a relaxed DNA topology. All *C*. *jejuni* strains were cultured on Mueller Hinton (MH) agar (Oxoid) at 37 °C under microaerophilic conditions generated using Campygen gas packs (Oxoid). For liquid cultures, *C*. *jejuni* strains were equalized to specific optical densities in MH broth and incubated under microaerobic conditions at 37 °C, shaking at 200 rpm. *C*. *jejuni* stock cultures were maintained using MH broth (Oxoid) supplemented with 20% glycerol and stored at −80 °C. Strains carrying the pRY107 plasmid were grown in the presence of 50 μg/ml kanamycin to ensure maintenance of the plasmid. To relax DNA supercoiling levels strains were grown in the presence of 10 μg/ml novobiocin.

### Selection for fluoroquinolone resistant mutants

NCTC11168 WT or NCTC11168 containing pRY107 were streaked on to MH agar plates supplemented with increasing concentrations of ciprofloxacin or nalidixic acid with an aim to isolate colonies resistant to each antibiotic using a method adapted from previous similar studies^33,34^. In each case, a single colony was selected and restreaked on to a MH agar plate containing a higher concentration of fluoroquinolone. The concentration increments were as follows 10, 25, 50, 75,100 μg/ml nalidixic acid and 5,10, 25, 50 μg/ml ciprofloxacin. The guideline of 100 μg/ml nalidixic acid resistance and 50 μg/ml ciprofloxacin was determined from the NARMS: Human isolates final reports information on *Campylobacter* isolate antimicrobial resistance (CDC, 2018, table 46). Once the natural mutants were generated, the NCTC11168 FQ^R^ mutants were subsequently grown in the same antibiotic-free conditions as the WT NCTC 11168. All FQ^R^ mutants were shown to grow at similar rates to the parent NCTC 11168 (Supplementary Fig. 1).

### Fluorescence assay for visualisation of biofilm

Overnight cultures were equalised to an OD600 of 0.1 in MH broth and 200 μl was seeded in triplicate into the wells of a PerkinElmer CellCarrier ultra optical 96 well plate. These were then incubated for 72 h at 37 °C microaerobically (5% O2) or aerobically (21% O2). In order to remove non-adherent bacteria the wells were then washed 3 times with PBS (Sigma). The metabolically active bacteria were then stained for 30 mins with PBS containing 40 μg/ml 5-TAMRA-SE (5-Carboxytetramethylrhodamine, Succinimidyl Ester, single isomer, ThermoFisher Scientific) viable stain. This rhodamine based dye is cell permeant and upon entering the cytosol is enzymatically crosslinked by bacterial esterases preventing the rhodamine dye from escaping the intact bacterial cell, providing a bright indicator of living bacteria. Upon staining with TAMRA, the wells were washed with PBS a further 5 times followed by fixing with 4% PFA for 10 minutes. Counterstaining of dead bacteria and extracellular DNA structures was carried out by staining the wells with PBS containing 10 μg/ml SytoX green dye for a further 30 minutes, followed by 3 additional PBS washing steps. Widefield imaging was carried out using a Leica DMI6000 Inverted epifluorescence microscope (Leica, Wetzlar, Germany). Images were acquired using a 5x N Plan 0.12NA air immersion objective at a resolution of 1600 × 1024 pixels. Confocal imaging was carried out using a FluoView FV1000 laser scanning microscope (Olympus, Tokyo, Japan). Images were acquired with a 60x UPLSAPO 1.35NA (Olympus, Tokyo, Japan) oil immersion objective at a resolution of 1024 × 1024 pixels in sequential scanning model acquiring images for channels 488 nm (SytoX) and 559 nm (TAMRA).

### Chloroquine gel electrophoresis to visualize *in vivo* plasmid DNA supercoiling profiles

Chloroquine gel electrophoresis was carried out on pRY107 plasmids isolated from 81-176, NCTC 11168 WT as well as nalidixic acid and ciprofloxacin mutants. Plasmid isolation was carried out on 20 ml of overnight cultures using the QIAgen MiniPrep kit and DNA concentrations were normalized using a NanoDrop spectrophotometer. All chloroquine gels comprised of 1% agarose made with 2X Tris borate EDTA (TBE) buffer. Addition of 10 μg/ml chloroquine salt solution to both the gel and running buffer allows relaxed circular DNA to migrate further while supercoiled circular DNA is retarded in the gel and remains higher. All chloroquine gels were run for 24 h at 100 V. At this point the gel was washed by immersion in ddH20 over 8 hours with water changes ever 15–30 minutes, followed by overnight washing to remove the chloroquine salt. Ethidium bromide solution at a concentration of 10 μg/ml was then added for two hours to visualize plasmid DNA.

### Measurement of bacterial motility

Each strain was inoculated into the centre of a 30 ml motility agar plate (MH broth containing 0.3% agar) using a 200 μl sterile pipette tip. Each plate was incubated upright for 48 h, after which it was imaged and the halo diameter was measured. Three biological replicates were carried out for each strain. The motility for each experiment is calculated as a percentage of the diameter of the agar plate.

### Culture of *in vitro* cell lines

The human ileocecal adenocarcinoma HT29 cell line was purchased from the (ATCC, HTB- 38) and chosen for its suitability as an intestinal invasion model and for uniformity in cell size and monolayer growth. HT29 cells were grown using McCoy’s 5A medium (Gibco) supplemented with 10% heat inactivated FBS. Cells were incubated at 37 °C with 5% CO_2_ in a humidified atmosphere 75 cm^2^ tissue culture flasks. The cells were routinely subcultured at roughly 80% confluency for no more than 10 passages.

### Fluorescence based invasion assay and differential staining

For invasion assays, 5 × 10^4^/cm^2^ cells were seeded onto 12 well plates (Corning) containing 16 mm optical coverslips, for 10 days, changing the media every 2 days. The incubation time allows for a tightly organised monolayer to cover 90–100% of the well, resulting in a more accurate epithelial monolayer model and for more consistent interactions between the monolayer barrier and invading bacteria.

Strains were grown on MH agar for 2 days in microaerophilic conditions then equalized at an O.D.600 of 0.02 in MH broth and incubated for 18 h at 37 °C, shaking at 200 rpm under microaerophilic conditions. The strains were then viability stained with TAMRA by Centrifuging at 4000 rpm for 10 mins and resuspending the bacteria in PBS containing 40 μg/ml TAMRA and incubating microaerophilically for 30 minutes, shaking at 200 rpm. The TAMRA labelled strains were then washed by 4 cycles of centrifugation and resuspension in PBS. In order to obtain a multiplicity of infection of 100:1, the vital stained strains were then equalised to an OD600 of 0.2 in McCoy’s 5A media supplemented with 10% heat inactivated FBS and invaded on to the HT29 monolayer for 3 h. The wells were then washed 3 times with PBS and the cells fixed for 15 mins with 4% PFA. For immunofluorescence double staining of the external bacteria, The cells were not permeabilised as the strains were previously vital stained (red) with TAMRA and cell impermeable immunofluorescence double staining of the external bacteria with a green secondary antibody allowed for visual distinction between adherent *C*. *jejuni* and fully internalised. A blocking solution of PBS containing 10% goat serum (Sigma) and 1% BSA (Sigma) was placed in the wells for 1 hour. A 1:200 dilution of polyclonal Rabbit-anti-*C*. *jejuni* antibody^35^ in PBS containing 1% BSA was then incubated with the cells for 45 mins followed by 3 washes with PBS. A 1:400 dilution of goat-anti-rabbit-Alexa488 was then incubated with the cells for 30 minutes followed by another 3x washes with PBS. The nuclei of the HT29 cells were then stained by 1:5000 dilution of cell permeable Hoechst33342 for 10 minutes. Cells were then washed 3x with PBS and stored in PBS.

### Gentamicin protection assay

In order to quantify the internalised bacteria after a 3 h invasion, invasion assays were carried out as described previously^23^ with the following alterations. After 3 h, the media overlying the infected HT29 cells was changed to complete McCoy’s 5A medium supplemented with 200 μg/ml gentamycin sulphate (Lonza) and infected HT29 cells were incubated at 37 °C under microaerophilic conditions for a further 2 h. After this, cells were washed 3 times with PBS and lysed with 1% Saponin in PBS for 15 min. Serial dilutions of the cell lysates were carried out using MH broth and plated on MH agar. All MH plates were incubated for 48 h under microaerophilic conditions using Campygen gas packs (Oxoid) at 37 °C followed by counting of colony forming units.

### *Galleria mellonella* larvae *in vivo* model of infection

Galleria invasion was carried out as previously described^36^. Sixth-instar larvae of *G*. *mellonella* were acquired from Livefoods Direct and stored in wood shavings in an incubator maintained at 15 °C. 70 larvae (0.2–0.4 g each) were randomly chosen for each strain assayed. Bacterial cells were grown microaerobically in MH broth for 24 hour and harvested by centrifugation at 4000 × g for 10 min and washed in PBS. The OD600 was obtained and cultures were diluted to a standard of 1.0 OD600 in PBS. Bacterial cultures were then serially diluted to 10^−7^. The bioburden per 20 μl inoculum was calculated post injection by plate counts of dilutions from 10^−4^ to 10^−7^, plated in triplicate. Ten larvae were inoculated with 20 μl each dilution through the last right pro-leg into the haemocoel using a 0.3 ml Myjector syringe (Terumo Europe) and incubated in Petri dishes, on filter paper, at 37 °C for 72 h. The control group consisted of ten larvae injected with 20 μl sterile PBS. If more than one larva died in the control group the experiment was discarded. Larval death was followed for 72 h, at 24 h intervals, by visual inspection and by the lack of movement when stimulated. Three independent trials were conducted consisting of ten larvae per bacterial concentration for each specified strain. Results are shown as survival over time. Lethal dose 50% (LD50) values were calculated using GraphPad Prism v.6.01 (GraphPad Software, La Jolla California USA). A log(inhibitor) vs. response curve was utilised to calculate the LD50 for each strain at 24 h, 48 h and 72 h post inoculation and averaged over three replicates.

### Expression and purification of *Campylobacter* gyrase subunits

A range of expression vectors containing the *C*. *jejuni* WT *gyrA* gene, *gyrB* gene and *gyrA* genes possessing the point mutations corresponding to Thr86Ile, Thr86Ala, Thr86Lys, Asp90Asn, and Asp90Tyr were provided by the laboratory of Prof. Suzuki and their construction has been previously described^37^. Recombinant *Campylobacter* gyrases were expressed and purified using this previously described methodology. In summary, the vectors containing the *Campylobacter* gyrases were transformed into *E*. *coli* BL21 (DE3/pLysS). Selected transformants were grown at 37 °C in LB broth supplemented with 100 μg/ml Ampicillin until an OD590 of 0.6 was reached. Recombinant GyrA and GyrB expression was subsequently induced by the addition of 1 mM of isopropyl β-D-thiogalactopyranoside (Wako Pure Chemicals Industries, Ltd.) and the culture was incubated at 18 °C for 16 h, shaking at 200 rpm.

Recombinant *E*. *coli* were then sonicated on ice at a 30% cycle, consisting of 10 cycles of 40s on and 40s +off (Sonifier 250; Branson, Danbury, CT, USA). After centrifugation (6000 × g for 15 min), recombinant DNA gyrase subunits in supernatants were purified by column chromatography using Ni-NTA agarose resin and dialysed against DNA gyrase dilution buffer (50 mM Tris–HCl, pH7.5; 100 mM KCl; 2 mM DTT; 1 mM EDTA). The protein fractions were examined by sodium dodecyl sulfate-polyacrylamide gel electrophoresis (SDS-PAGE) with Prestained Protein Marker, Broad Range (7–175 kDa) (New England Biolabs).

### *In vitro* DNA supercoiling assay

The DNA supercoiling activity of recombinant DNA gyrases was assayed *in vitro*. This involved measuring the ability of the gyrase AB complexes to supercoil the relaxed pBR322 plasmid over time. Supercoiling activity was compared between WT GyrA subunit and the 5 known GyrA mutants. In all reaction mixtures, WT GyrB protein was used to form the heterotetramer.

As described previously^37^, The reaction mixture consisted of DNA gyrase assay buffer (35 mM Tris–HCl pH7.5, 24 mM KCl, 4 mM MgCl_2_, 2 mM DTT, 1.8 mM spermidine, 1 mM ATP, 6.5% glycerol, and 0.1 mg/mL of BSA), 0.3 μg of relaxed pBR322 DNA, and 36 nM of GyrA and GyrB in a total volume of 30 μl. Reactions were run at 37 °C for a time course of 5, 10, 15, 30, 45, 60, 120, 150 minutes and stopped by the addition of 8 μl of stop solution (5% SDS, 25% Glycerol and 0.25 mg/ml Bromophenol blue) as previously described^38^. Supercoiling of pBR322 was measured by loading 10 μl of each reaction mixtures into wells of 1% agarose gel in Tris-borate-EDTA (TBE) buffer as well as DNA ladder for quantification of bands. Electrophoresis was carried out for 120 mins at 40 mA and stained with ethidium bromide (0.5 μg/mL). The extent of supercoiled DNA was quantified by densitometry analysis with FiJi imageJ software (http://rsbweb.nih.gov/ij) compared to DNA samples of known concentration.

### Statistical analysis

Tests for statistical analysis were calculated using the unpaired student’s t-test (two tailed distribution) with Welch’s correction. Statistically different values possessed *P*-values of ≤0.05. Data is representative of a minimum of three biological replicates per condition. Error bars represent the standard deviation for each sample condition.

## Results

### Resistance to fluoroquinolones in *C*. *jejuni* confers the ability to form a viable biofilm under aerobic conditions

To test whether a correlation existed between fluoroquinolone resistance and biofilm formation a panel of natural FQ^R^ mutants were selected for by plating strain NCTC11168 on increasing concentrations of the fluoroquinolone ciprofloxacin or the closely related quinolone antibiotic nalidixic acid. Multiple strains were isolated with MIC values of 50 μg/ml and 100 μg/ml respectively. These strains were then incubated in the absence of antibiotics and their ability to generate viable biofilms measured in comparison to the wild type sensitive strain. Strains were incubated for 72 hours under microaerobic and aerobic conditions and stained with TAMRA to allow the visualization of viable bacteria within the biofilm.

**Figure 1:**
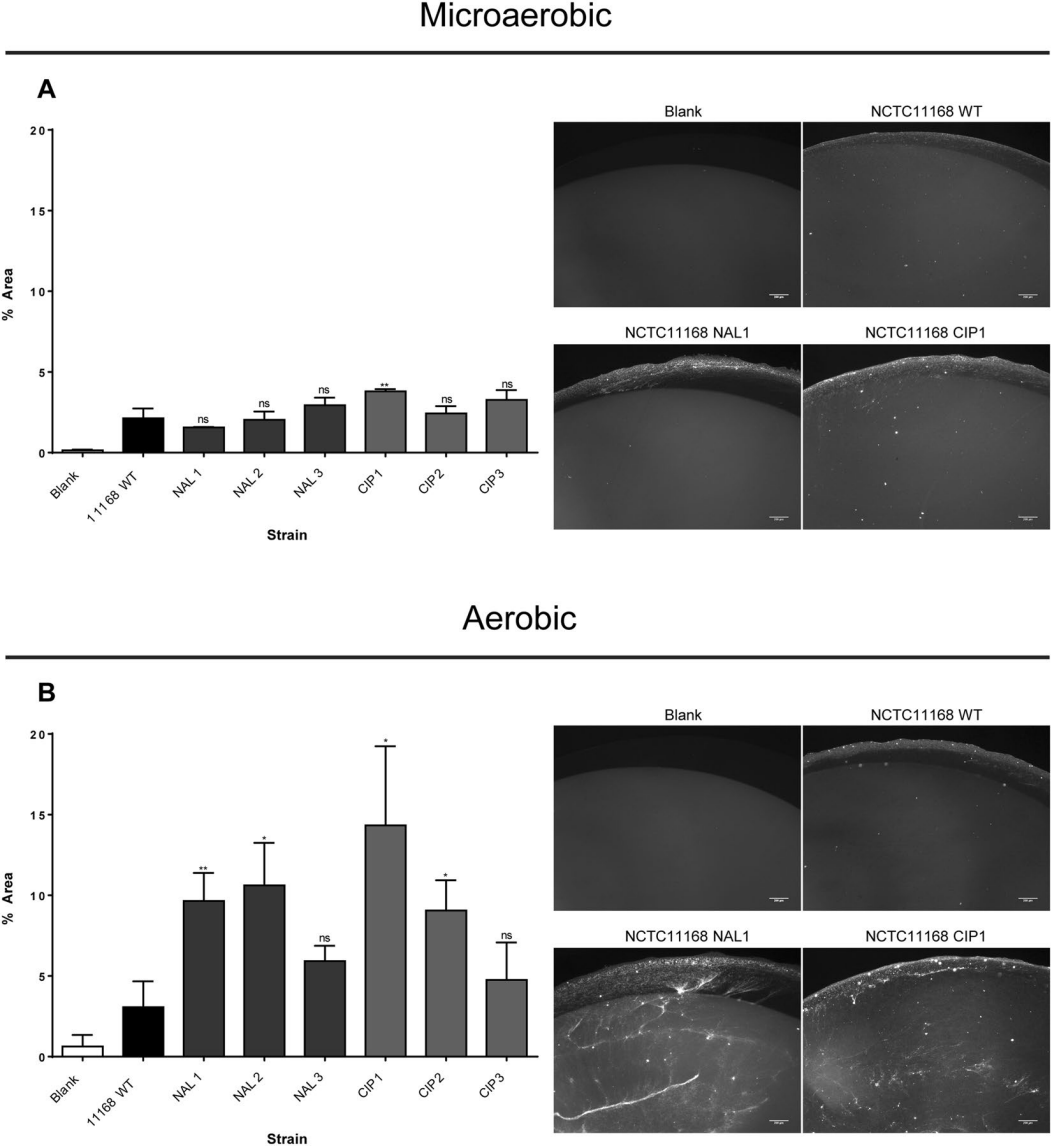
Quantification of TAMRA stained viable adherent biofilm formation for WT NCTC11168, nalidixic acid resistant and ciprofloxacin resistant mutants after 72 h grown.

As shown in Fig. 1A very little difference could be seen between the wildtype strain and the mutants resistant to either antibiotic tested under microaerobic conditions with a minimal amount of viable bacteria evident. However under aerobic conditions (Fig. 1B) an increase in viable biofilm formation was observed in all the mutants resistant to both ciprofloxacin and nalidixic acid with most showing large statistically significant increases in numbers of viable bacteria, suggesting that the mutations which conferred resistance also conferred a greater ability to form a viable biofilm in the presence of atmospheric levels of oxygen. This increase in biofilm formation was not due to an increase in growth as no significant difference was observed between the wildtype strain or mutants in overnight growth under either microaerobic or aerobic conditions (Supp Fig. 1)

### Mutants resistant to fluoroquinolones display an altered DNA supercoiling profile

**Figure 2:**
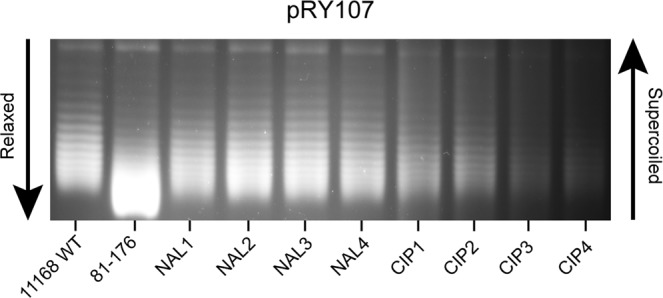
Chloroquine gel electrophoresis of pRY107 from WT and fluoroquinone resistant strains reveals a shift in *in vivo* supercoiling profile towards a relaxed phenotype for NCTC11168 with either nalidixic acid or ciprofloxacin resistance.

Since fluoroquinolone resistance has previously been associated with changes in DNA supercoiling we tested the supercoiling profiles of naturally acquired FQ^R^ mutants with the wild type 11168 strain and a strain with a much more relaxed DNA topology (81-176). Strains resistant to naladixic acid displayed a more relaxed DNA supercoiling phenotype with an increase in more relaxed topoisomers visible migrating further down the gel (Fig. 2). Strains resistant to ciprofloxacin showed a greater variation with some strains displaying a more relaxed phenotype similart to the naladixic acid mutants while others displayed a DNA topology indistinguishable from the wildtype. This observation led us to postulate as to whether the biofilm phenotype observed in Fig. 1 was mediated by changes in resting DNA supercoiling levels of the FQ^R^ mutants.

### Relaxation of DNA Supercoiling leads to an increase in viable biofilm formation in wild type strain NCTC11168

**Figure 3:**
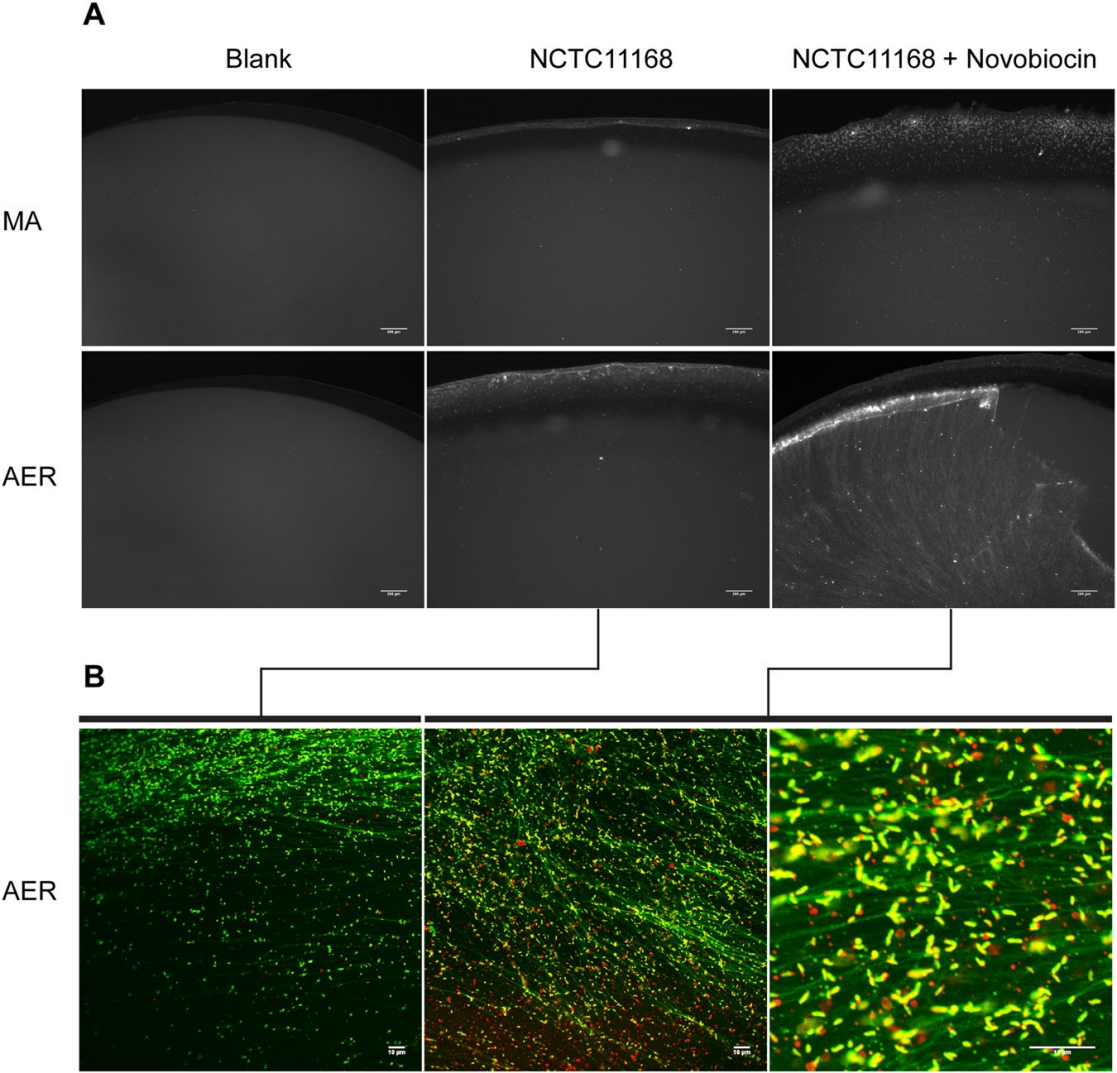
(**A**) 72 h biofilm assays were carried out on Cellcarrier Ultra 96 well plates followed by staining of viable *C*. *jejuni* with the vital stain TAMRA for 30 minutes. For NCTC11168 a stark increase in the formation of a mature, viable biofilm is present in aerobic conditions (AER) but not microaerobic conditions (MA) when a relaxed DNA supercoiling phenotype is induced by growth in the presence of 10μg/ml Novobiocin. Data is representative of three biological replicates. (**B**) Confocal slices of TAMRA (live bacteria, red) and SytoX (DNA, green) stained induced biofilm after 72 h incubation in aerobic conditions. Relaxation of DNA supercoiling results in an increase in both viable NCTC 11168 as well as fibers of eDNA, indicating a mature *C*. *jejuni* biofilm (scale bar = 10 μm).

We have previously shown that growth in sub-inhibitory concentrations of novobiocin can be used to dose dependently relax DNA supercoiling in strain NCTC11168. Using this approach the ability of *C*. *jejuni* strain NCTC 11168 to form viable biofilm upon DNA relaxation was tested^23,24^. Growth in the presence of novobiocin led to an increase in the amount of viable biofilm formed (Fig. 3A). This increase was much more pronounced under aerobic conditions where relaxation of DNA supercoiling by growth in the presence of novobiocin appeared to dramatically increase the ability of the bacteria to form a viable biofilm.

To further characterise this induced biofilm the cells were stained with a combination of the vital stain TAMRA to stain viable bacteria and SytoX to stain extracellular DNA (eDNA) and analysed using confocal microscopy. An increase in viable (red) bacteria was observed in the biofilm created by the bacteria which were treated with novobiocin to relax DNA supercoiling, as opposed to the biofilm formed by untreated bacteria (Fig. 3B). In addition an increase in eDNA fibers could be observed suggesting that the biofilm is similar to those previously described for *C*. *jejuni*.

### Fluoroquinolone resistance is associated with reduced motility and increased invasion of epithelial cells

**Figure 4:**
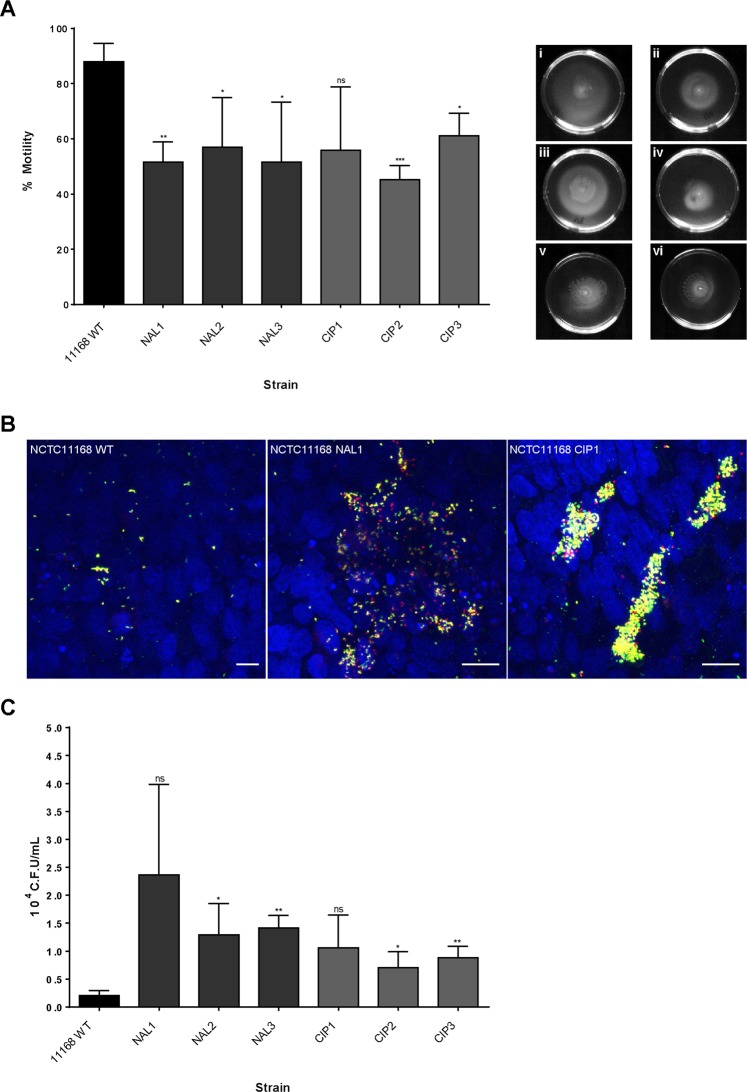
(**A**) Three biological replicates of percent motility in 0.3% MH Agar of WT 11168 and 11168 derived FQ^R^ mutants at 48 h (P > 0.05). (i) NCTC11168 WT (ii) NCTC11168 NAL1. (iii) NCTC11168 NAL2. (iv) NCTC11168 NAL3. (v) NCTC11168 CIP2. (vi) NCTC11168 CIP3. (**B**) Immunofluorescence confocal maximum Z-projections of TAMRA stained NCTC 11168 WT and two FQ^R^ strains (Internalised, red. External, yellow) invading a HT29 monolayer after 3 h (scale bar = 10μm). A large increase in adherent *C*. *jejuni* was evident for FQ^R^ strains in comparison to the WT. (**C**) The gentamycin protection assay was used to quantify invasion via C.F.U. of internalised *C*. *jejuni* after 3 h incubation onto HT29 monolayers. A trend of increased invasion was evident for all 6 FQ^R^ strains (P > 0.05).

To confirm whether changes in resting DNA supercoiling levels in FQ^R^ strains were responsible for changes in phenotypic behaviour the strains were analysed for changes in phenotypes which have been previously shown to be altered by changes in DNA supercoiling. We have previously reported that relaxation of DNA supercoiling leads to a decrease in motility and an increase in invasion of epithelial cells in *C*. *jejuni*^23,24^. FQ^R^ mutants were less motile than the WT 11168 strain when grown in the absence of antibiotics (Fig. 4A), which would indicate that the more relaxed DNA supercoiling observed in these strains has reduced their motility. In addition these less motile FQ^R^ strains invaded epithelial cells in higher numbers than the wildtype strain as observed by immunofluorescence and by the gentamycin protection assay (Fig. 4B,C) as we have previously reported for strains whose DNA supercoiling has been altered by treatment with novobiocin or with naturally more relaxed DNA supercoiling.

The FQ^R^ strains displayed a phenotype by immunofluorescence which revealed increased numbers of bacteria adherent to the epithelial cell surface. The Ciprofloxacin mutants in particular displayed a phenotype with large aggregates of bacteria interacting with the epithelium at specific points. The more relaxed resting levels of DNA supercoiling observed in these FQ^R^ mutants clearly resulted in a much more invasive phenotype raising the question as to whether these mutants would be more virulent *in vivo*.

### Fluoroquinolone resistance leads to increased virulence in the *in vivo* model *Galleria mellonella*

To investigate whether the changes in DNA supercoiling observed in the FQ^R^ mutants could actually lead to an increase in the ability of these strains to cause disease a larval survival assay was employed. Larvae were challenged with different concentrations of the *C*. *jejuni* WT and fluoroquinolone resistant strains to investigate the kinetics of virulence for each strain. Larval death as well as larval melanisation caused by individual strains at each concentration was then recorded at different timepoints.

**Figure 5:**
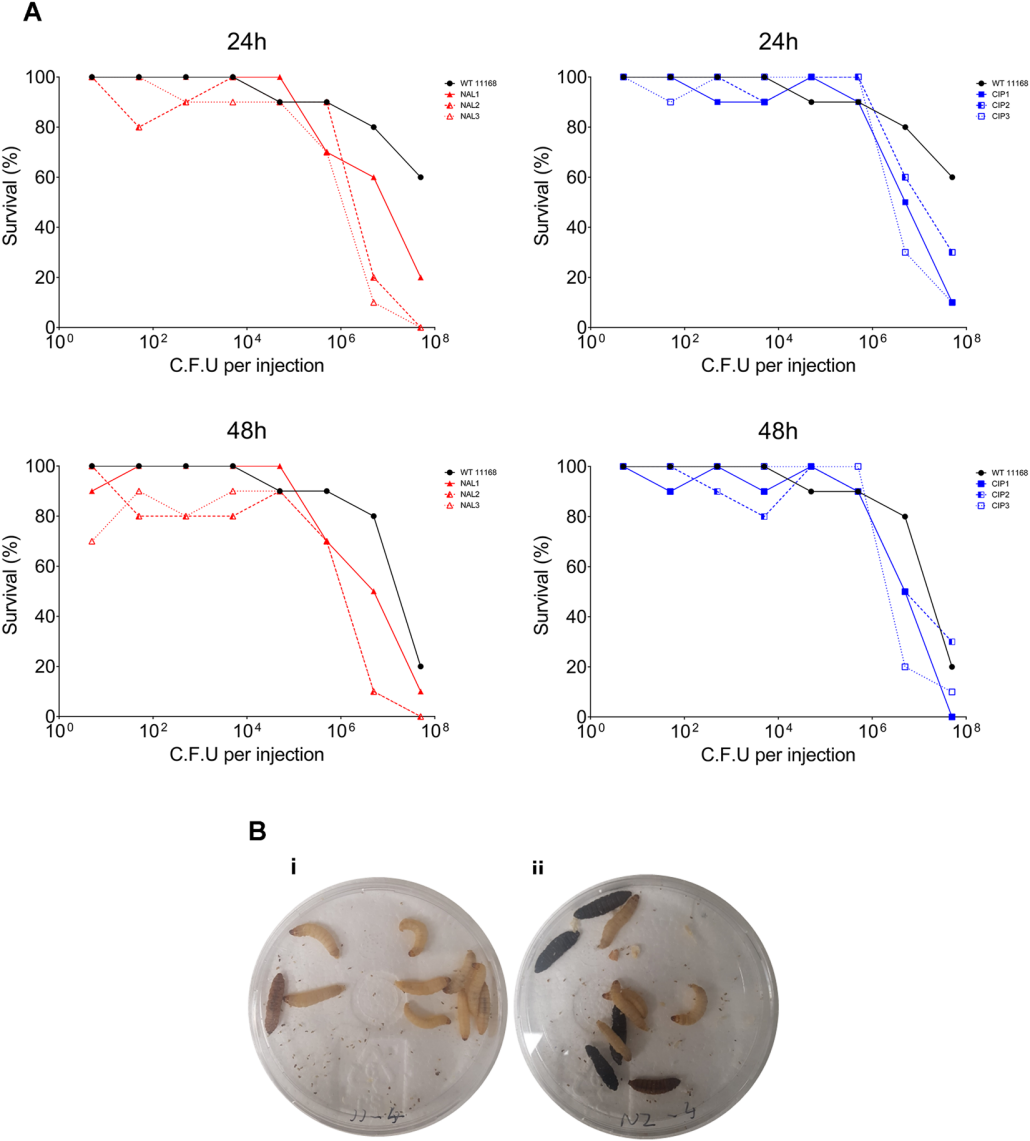
(**A**) Kinetics of *Galleria mellonella* larval killing by *C*. *jejuni* NCTC 11168 and NCTC11168 strains resistant to nalidixic acid and ciprofloxacin. Survival of *G*. *mellonella* at 24 h or 48 h following infection with *C*. *jejuni*. Each dataset is from a single trial (N = 10) which was representative of three independent replicates for each strain. The data are presented as percentage larval survival from ten larvae injected per trial. No control larvae died in any given trial. (**B**) *Galleria mellonella* 48 h post infection challenge with 10^3^ (i) NCTC11168 WT and (ii) NCTC11168 NAL2.

All FQ^R^ mutants tested displayed a greater larval killing efficiency at 24 hours post infection than the WT strain NCTC11168 (Fig. 5A and Table 1) with many mutants capable of killing larvae at inocula as low as 10^2^ cfu/ml. The difference in killing efficiency between the mutants and the wildtype was particularly pronounced between inocula of 10^6^–10^8^ cfu/ml after 24 hours. Melanisation, which is a phenotype associated with an increased immune response and involves melanin depositing around pathogens, was only ever observed in larvae infected with FQ^R^ strains (Fig. 5B).

**Table 1:** Log LD_50_ of *G*. *mellonella*.

Strain	24 h	48 h	72 h
NCTC11168	7.59 ± 0.02	7.24 ± 0.26	6.62 ± 0.25
NAL1	6.60 ± 0.08**	6.22 ± 0.20*	6.01 ± 0.06^ns^
NAL2	6.25 ± 0.04****	6.06 ± 0.03*	6.03 ± 0.02^ns^
NAL3	6.01 ± 0.06***	5.97 ± 0.20**	5.90 ± 0.19*
CIP1	6.36 ± 0.22*	6.34 ± 0.23*	5.90 ± 0.34^ns^
CIP2	6.05 ± 0.04****	5.95 ± 0.05*	5.91 ± 0.06^ns^
CIP3	6.49 ± 0.03****	6.44 ± 0.10*	6.23 ± 0.17^ns^

LD50 was calculated for each strain LD50 for each strain at 24 h, 48 h and 72 h post inoculation and averaged over three replicates. Data is expressed as mean Log LD50 (±SD) (n = 3). Statistically significant differences were calculated using the Welch two sample t-test, * denotes p ≤ 0.05.

### Identification of mutations involved in fluoroquinolone resistance and confirmation of their role in altering DNA supercoiling levels

To identify the mutations in our FQ^R^ mutants the strains were whole genome sequenced and their genomes analysed for mutations in sites known to be associated with fluoroquinolone resistance. No mutations were observed in *cmeA*, *cmeB* or *cmeC* which encode for a drug efflux pump (Table 2). All strains resistant to nalidixic acid tested had the previously reported FQ^R^ resistant conferring mutation Thr86Ile. All three ciprofloxacin-resistant strains also had this mutation but two had an additional Asp90Asn mutation in *gyrA* and the other had a Leu458Lys mutation in *gyrB*.

**Table 2:** FQ^R^ conferring mutations.

Strain	Amino acid substitutions					
	*gyrA*	*gyrB*	*topA*	*cmeA*	*cmeB*	*cmeC*
NCTC11168	—	—	—	—	—	—
NAL1	Thr86Ile	—	—	—	—	—
NAL2	Thr86Ile	—	—	—	—	—
NAL3	Thr86Ile	—	—	—	—	—
CIP1	Thr86Ile, Asp90Asn	—	—	—	—	—
CIP2	Thr86Ile, Asp90Asn	—	—	—	—	—
CIP3	Thr86Ile	Leu458Lys	—	—	—	—

**Figure 6:**
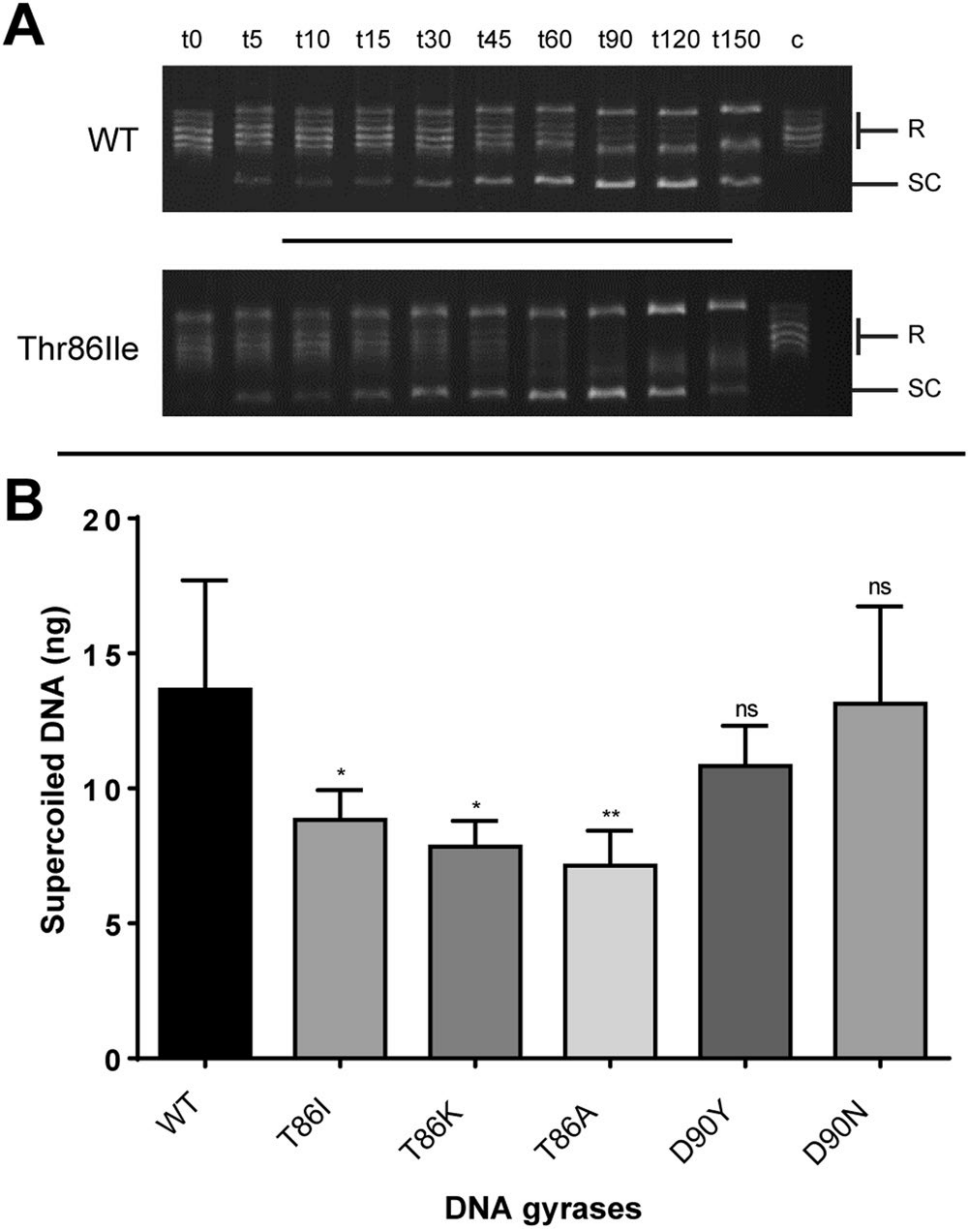
Wild type *C*. *jejuni* gyrase A and gyrases possessing all known FQR point mutations were expressed and their ability to supercoil relaxed plasmid DNA over time was determined via *in vitro* supercoiling assay. Relaxed 0.3 μg pBR322 was incubated in the presence of 36 nM each of WT GyrB and WT GyrA or GyrA possessing Thr86Ile, Thr86Lys, Thr86Ala, Asp90Tyr and Asp90Asn. The reactions were then stopped at intervals from time: 0 to time: 150 mins and the reaction products were analysed via electrophoresis on 1% agarose. (**A**) Representative images of supercoiling assay for WT GyrA and Thr86Ile GyrA visualising he proportion relaxed (R) pBR322 plasmid converted to supercoiled DNA (SC) at each timepoint (t). Lane C contains relaxed pBR322 plasmid, lanes *t*0–*t*150 contain reaction mixture in which the reaction was stopped at the timepoint denoted by *t*. (**B**) Supercoiled plasmid (SC) was quantified via densitometry analysis using DNA standards of known concentration with *t*60 being represented in this graph. The predominant mutant Thr86Ile exhibited statistically lower supercoiling activity (p ≤ 0.05) indicating this common mutant could shift the supercoiling profile of FQR strains, conferring a phenotype promoting virulence.

To test whether the Thr86Ile mutation was sufficient to affect the resting DNA supercoiling levels an *in vitro* assay was carried out testing the ability of recombinant GyrA with different mutations to change DNA supercoiling levels. Mutation of Thr86 resulted in a significant decrease in the supercoiling levels of DNA (Fig. 6) suggesting that this mutation was sufficient to relax DNA supercoiling and confer these secondary phenotypes promoting virulence.

## Discussion

In recent decades a dramatic increase in antimicrobial resistance in human pathogens has been identified as being a key threat to human health by the World Health Organization^8–10^. Since the 1980s reports have highlighted a trend of increased fluoroquinolone resistance in *Campylobacter jejuni* strains isolated from clinical and livestock sources in different countries^8,39^. Although this rise in strains resistant to fluoroquinolones has been studied and the mechanism of resistance in strains has been well characterised it is still poorly understood how this acquisition of resistance affects other phenotypes. Other human pathogens such as *Pseuodomonas aeruginosa* and *Staphylococcus aureus* have been shown to have altered virulence gene expression upon acquisition of antibiotic resistance^40–43^. One study has shown that *C*. *jejuni* strains resistant to fluoroquinolone mutations have a fitness advantage over fluoroquinolone-sensitive strains during the commensal colonization of chickens^17^. These fluoroquinolone mutants have mutations in the *gyrA* gene that encodes for one half of the GyrAB heterodimer which is central in regulating DNA supercoiling. Given our recent reports of the critical role played by DNA supercoiling in the regulation of virulence in *C*. *jejuni*^23,24^ we set about to investigate whether the acquisition of fluoroquinolone resistance would have an effect on a variety of phenotypes.

To investigate any changes in phenotype a panel of mutants were created to the commonly used fluoroquinolone ciprofloxacin and the closely related quinolone nalidixic acid by selection on increasing concentrations of these antibiotics as previously described. These individual mutants were then compared for their ability to form biofilm using a microscope based assay staining with the TAMRA viable stain. Although no significant increase in biofilm formation was observed under microaerobic conditions, under aerobic conditions the FQ^R^ mutants all formed a much greater biofilm than that observed in the wildtype. Most resistant clones had a more relaxed resting DNA supercoiling level than the wildtype strain and this observation agrees with a previous study which associated fluoroquinolone resistance with changes in DNA supercoiling^18^. To further prove a correlation between DNA supercoiling and biofilm formation it was shown that biofilm formation could be induced by artificially relaxing resting DNA supercoiling levels using subinhibitory levels of novobiocin. The fact that the biofilm was much more pronounced under aerobic conditions than under microaerobic conditions, suggests that changes in DNA supercoiling are essential but that as previously reported oxidative stress also plays a key role^5,29^. Although further work studying the supercoiling profiles of viable bacteria within aerobic biofilms will be needed to properly understand the interplay between DNA topology and oxidative stress. Interestingly in our previous study of the secretome induced under relaxed DNA supercoiling some of the most notable proteins were those involved in oxidative stress such as KatA and SodB^23^. This raises the prospect that the same secretome which induced invasion of epithelial cells may also be useful in creating a viable biofilm under aerobic conditions. The induction of a viable biofilm in the presence of oxygen by these antibiotic resistant strains could offer a significant advantage in their ability to survive key environments which allow for transmission of the bacteria. The nature of the biofilm formed was interesting as it appeared to include large numbers of non-viable bacteria and what appeared to be an extracellular matrix of eDNA. This would agree with previous reports which have implicated eDNA in the formation of biofilms by *C*. *jejuni*^44,45^.

Having established that acquisition of fluoroquinolone resistance induced relaxation of DNA supercoiling which in turn led to the ability to form greater biofilm under aerobic conditions we then tested whether these mutants were affected in other DNA supercoiling associated phenotypes. All mutants were shown to have an increased ability to invade epithelial cells and a reduced motility. This observation concurs with our previously published studies which reported that strains with more relaxed DNA supercoiling are less motile but more invasive^23,24^. Interestingly the ciprofloxacin mutants displayed a phenotype with large aggregates of bacteria interacting with the epithelium and future studies need to investigate the significance of these aggregates. This increased invasion now raises the prospect that fluoroquinolone-resistant strains may not only be more likely to be transmitted to humans but may also be primed to be more invasive. To test this theory the resistant mutants and the WT were used to infect *Galleria mellonella* moth larvae which has been previously used as a model for *C*. *jejuni* invasion^46^. The results revealed that larvae infected with each of the antibiotic-resistant strains required far fewer bacteria in the initial inoculum to cause death than the WT strain suggesting a much more pathogenic phenotype.

To finally confirm that the altered supercoiling was being induced by mutations in *gyrA* the mutants were fully sequenced. All the strains tested contained the Thr86Ile mutation in *gyrA* which has been previously described as the most common mutation associated with fluoroquinolone resistance. Interestingly the ciprofloxacin mutants all had secondary mutations either in *gyrA* (Asp90Asn) or *gyrB* (Leu458Lys). This implicated the Thr86Ile mutation as the most likely to affect DNA supercoiling as it was found in all the strains tested and this was confirmed by an *in vitro* supercoiling assay where a Thr86Ile mutant GyrA was compared to the WT GyrA and shown to have deficient supercoiling ability in comparison to other GyrA mutations.

Overall the findings in this study reveal that acquisition of fluoroquinolone resistance in *C*. *jejuni* is associated with both an increase in viable biofilm formation under aerobic conditions as well as a more invasive phenotype *in vitro* and *in vivo*. Given the dramatic increase in fluoroquinolone-resistant strains in recent years, this may point to the fact that these resistant strains have an increased ability to survive in the external environment and thus transmit to new hosts. It is tempting to hypothesise that the prevalence of Thr86Ile as by far the most dominant FQ^R^ conferring amino acid substitution^12,47^, is not only due to the fact that the it confers high level FQR^15^ but that the disruption of Gyrase function, promotes relaxation of DNA supercoiling within a tolerable range, and in a coincidental fashion promotes the upregulation of genes sensitive to a relaxed supercoiling homeostasis. The upregulation of oxidative stress response and virulence genes by relaxation of DNA supercoiling could provide an alternate manner by which this FQ^R^ conferring trait may be selected for in strains in stress inducing environments even in the absence of FQ treatment, for example in the high oxygen environment of supermarket chicken meat. This could also explain previous reports that fluoroquinolone resistant strains of *C*. *jejuni* can thrive in poultry populations in the absence of Fluoroquinoone use and can persist in poultry populations long after the cessation of the use of these antibiotics^17,48,49^. Furthermore, the observation of a more invasive phenotype raises the prospect that these antibiotic resistant strains are also likely to be more pathogenic upon infection of humans. In conclusion the threat of fluoroquinolone resistance in *C*. *jejuni* appears not only to be the resistance to this class of antibiotics but also the effect of these mutations on phenotypes critical in the transmission and pathogenicity of these microorganisms.

## Supplementary information

Supplementary Fig 1
