# Supplementary material for: Acquisition of fluoroquinolone resistance leads to increased biofilm formation and pathogenicity in Campylobacter jejuni
Source: Sci Rep. 2019 Dec 3;9:18216. doi: 10.1038/s41598-019-54620-1 (PMC6890674; doi:10.1038/s41598-019-54620-1)
Supplement: Supplementary file 1 — Supplementary Fig 1 [file 41598_2019_54620_MOESM1_ESM.pdf]

Acquisition of fluoroquinolone resistance leads to increased biofilm formation and pathogenicity in *Campylobacter jejuni*.

Matthew V.X. Whelan[1], Laura Ardill[1], Kentaro Koide[2,3], Chie Nakajima[2,3] Yasuhiko Suzuki[2,3], Jeremy C. Simpson[4] and Tadhg Ó Cróinín[1]*

[1]School of Biomolecular and Biomedical Science, University College Dublin, Belfield, Dublin 4, Ireland

[2]Division of Bioresources, Hokkaido University Research Center for Zoonosis Control, Sapporo, Japan

[3]The Global Station for Zoonosis Control, Hokkaido University Global Institution for Collaborative Research and Education, Kita 20 Nishi 10, Kita-ku, Sapporo, Japan

[4]School of Biology and Environmental Science, University College Dublin, Belfield, Dublin 4, Ireland

Supplementary Figure 1

*Correspondence to Tadhg.OCroinin@ucd.ie

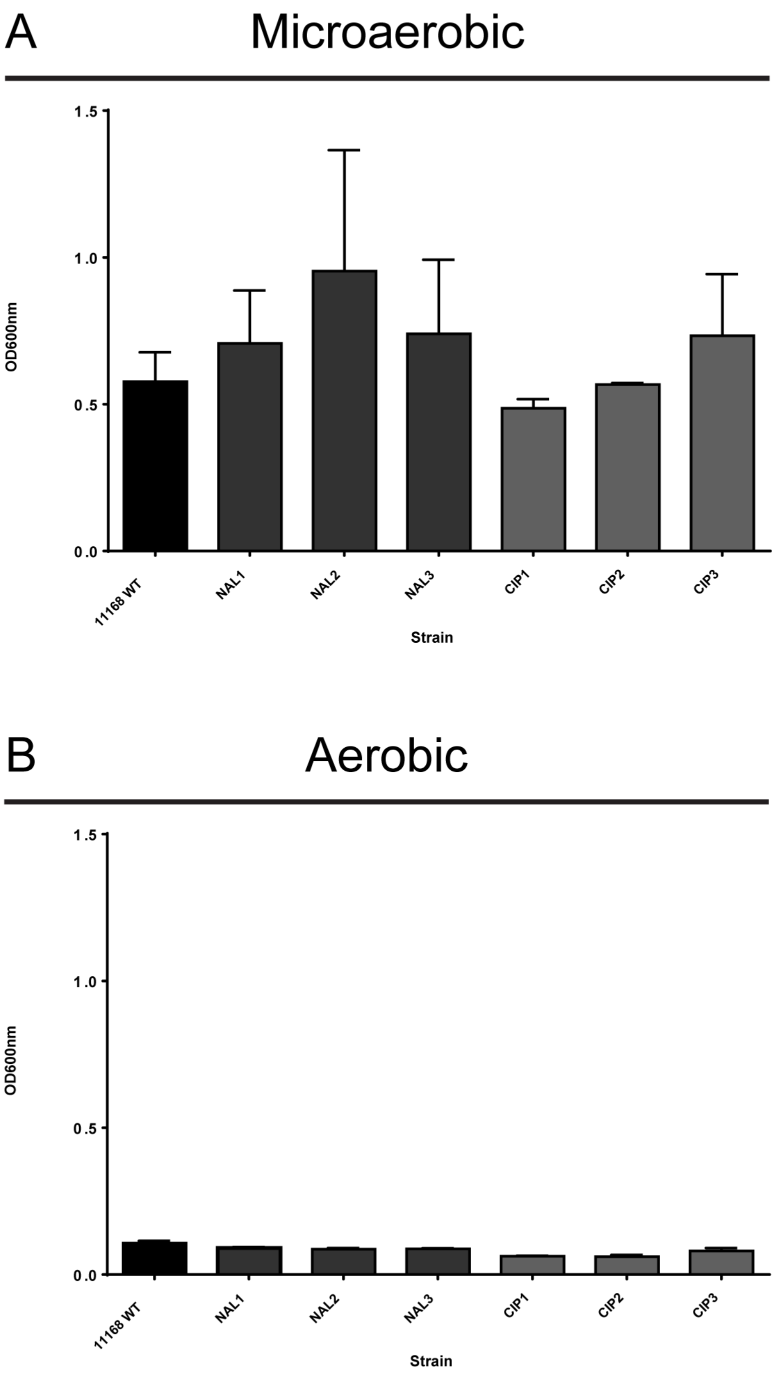
A
Microaerobic
1.5
1.0
0.5
0.0
OD600nm
11168 WT
NAL1
NAL2
NAL3
CIP1
CIP2
CIP3
Strain
B
Aerobic
1.5
1.0
0.5
0.0
OD600nm
11168 WT
NAL1
NAL2
NAL3
CIP1
CIP2
CIP3
Strain

Supplementary figure 1. Wildtype and FQ^R strains were grown microaerophilically and aerobically in MH broth for 24 hours and growth measured by optical density. No significant difference was observed between the Wildtype or any of the fluoroquinolone resistant strains. Data shown is mean +/- SD (n=3). Statistically significant differences were calculated using the Welch two sample t-test with no significance found for any condition in relation to the WT.
